# Higher-order Sonification of the Human Brain

**DOI:** 10.21203/rs.3.rs-6623643/v1

**Published:** 2025-06-26

**Authors:** Francisco-Shu Kitaura, Emi-Pauline Kitaura, Niels Janssen, Antonella Maselli, Ernesto Pereda, Aurelio Carnero Rosell

**Affiliations:** a Instituto de Astrofísica de Canarias, C/ Vía Láctea, s/n, E-38205, San Cristóbal de La Laguna, Spain; b Departamento de Astrofísica, Universidad de La Laguna (ULL), E-38206, San Cristóbal de La Laguna, Spain; c Facultad de Psicología, Universidad de la Laguna (ULL), E-38200, San Cristóbal de La Laguna, Spain; d Instituto de Tecnologías Biomédicas (ITB), Universidad de La Laguna (ULL), E-38200, San Cristóbal de La Laguna, Spain; e Instituto Universitario de Neurociencias (IUNE), Universidad de la Laguna (ULL), E-38200, San Cristóbal de La Laguna, Spain; f Institute of Cognitive Sciences and Technologies, National Research Council (CNR), Rome, Italy; g Departmento de Ingeniería Industrial, Universidad de la Laguna (ULL), E-38200, San Cristóbal de La Laguna, Spain

## Abstract

Sonification, the process of translating data into sound, has recently gained traction as a tool for both disseminating scientific findings and enabling visually impaired individuals to analyze data. Despite its potential, most current sonification methods remain limited to one-dimensional data, primarily due to the absence of practical, quantitative, and robust techniques for handling multi-dimensional datasets.

We analyze structural magnetic resonance imaging (MRI) data of the human brain by integrating two- and three-point statistical measures in Fourier space: the power spectrum and bispectrum. These quantify the spatial correlations of 3D voxel intensity distributions, yielding reduced bispectra that capture higher-order interactions. To showcase the potential of the sonification approach, we focus on a reduced bispectrum configuration which applied to the OASIS-3 dataset (864 imaging sessions), yields a brain age regression model with a mean absolute error (MAE) of 4.7 years. Finally, we apply sonification to the ensemble-averaged (median) outputs of this configuration across five age groups: 40–50, 50–60, 60–70, 70–80, and 80–100 years. The auditory experience clearly reveals differentiations between these age groups, an observation further supported visually when inspecting the corresponding sheet music scores. Our results demonstrate that the information loss (e.g., normalized mean squared error) during the reconstruction of the original bispectra, specifically in configurations sensitive to brain aging, from the sonified signal is minimal. This approach allows us to encode multi-dimensional data into time-series-like arrays suitable for sonification, creating new opportunities for scientific exploration and enhancing accessibility for a broader audience.

The technique of sonification has emerged as a valuable tool in various fields for its ability to transform complex data into auditory experiences^1^, making it easier for researchers and the public alike to grasp intricate concepts (e.g.,^2^). Beyond its role in disseminating scientific findings, sonification also serves as a crucial assistive technology for visually impaired individuals, allowing them to analyze data through auditory means (e.g.,^3, 4^). Sonification might even help revealing unrecognized patterns and feedbacks in unwieldy datasets^5^. A wide number of sonification methods and algorithms have been developed and applied in the context of astronomy^6–17^ and other fields (e.g.,^18, 19^). Recently, Artificial Intelligence algorithms based on Neural Networks and Deep Learning have also been implemented^20, 21^. Enge et al.^22^ present an overview of the field of sonification, analyzing the complementarity and redundancy between sonification and visualization. Recent studies even suggest that data sonification has emerged as a viable alternative to data visualization^23^.

It has been shown that audification as a tool for the spectral analysis of time-series data^24^ can be particularly useful in the presence of low signal-to-noise ratios, where smaller biases are obtained with auditory than with visual stimuli^25^. For long, it has been suggested to use the loudness, pitch, and duration of each instrumental tone to obtain a multi-dimensional perceptual scaling of musical timbres^26^. In fact, sonification can also be used to represent additional dimensions in complex multi-dimensional datasets^27–29^. Audification and sonification of texture in images, have also been proposed^30^ to represent spatial data, for example, in the context of geographical information^31, 32^, and biomedicine^33, 34^.

However, despite its growing popularity and utility as an additional tool to visualisation^35^, current sonification methods are primarily limited to one-dimensional data due to the lack of practical and robust methods for quantitatively handling multi-dimensional data with solely auditory stimuli. This limitation significantly restricts the range of applications and the depth of insight that sonification can provide. To address this gap, there is a need for innovative approaches that can effectively capture the complexity of multi-dimensional datasets and translate them into meaningful quantitative auditory representations.

One discipline poised to greatly benefit from sonification methods capable of handling multidimensional data is neuroimaging. The primary goal of this field is to extract meaningful information from complex 3D and 4D images that capture both the structure (3D) and dynamics (4D, with time as the fourth dimension) of the human brain, recorded through tomographic techniques such as X-ray computed tomography (CT), positron emission tomography (PET), or magnetic resonance imaging (MRI). In MRI, for instance, highly detailed 3D images of brain structures are obtained with excellent spatial resolution—typically using cubic voxels measuring 1×1×1 mm—where voxel intensity reflects the type and density of tissue. While these images are commonly used in clinical settings to detect abnormalities or injuries, advanced mathematical techniques in neuroimaging research allow for the quantification of these images and the identification of subtle differences between groups (e.g., healthy versus pathological subjects or across different age groups) or within the same individual over time. These differences are often not visually apparent due to the complexity of the 3D data, necessitating the use of sophisticated analytical tools to uncover them (see, e.g.,^36^). Sonification could offer an innovative approach to making these hidden patterns more accessible and interpretable.

In this study, as part of the CosmicBrain project, we propose a novel sonification method that leverages higher-order statistics in Fourier space to construct one-dimensional arrays from multi-dimensional data and apply it to human brain magnetic resonance imaging (MRI).

The analysis of three-dimensional spatial distributions is a well-established approach in cosmological large-scale structure studies, particularly for understanding the clustering properties of complex systems. In this context, we focus on using two-point and three-point correlation functions to probe the underlying structure of the data. The two-point function captures pairwise correlations, but the three-point function extends this to higher-order statistics. It was first computed in configuration space in the 1970s from the three-dimensional galaxy distribution^37^, and later extended to Fourier space as the bispectrum^38^. The three-point statistics are precious in cosmological studies for understanding structure formation^39^ and for investigating the bias between galaxies and dark matter tracers^40^ as it is sensitive to non-Gaussianities induced by gravitational evolution. These statistics are sensitive to the complex, multidimensional patterns in the data, capturing information beyond what two-point statistics can reveal^41^. By combining two- and three-point statistics, we gain a more comprehensive understanding of the clustering properties of structures, an approach that has been successfully applied to galaxy survey data (see^42^). In particular, the three-point function measures non-Gaussian features that are key for understanding the cosmic web’s morphology^39^.

Using three-dimensional MRI data, our study leverages these well-established statistical tools to characterize the clustering of brain structures. This is part of the CosmicBrain project, where the same techniques used in cosmology have been adapted to analyze the human brain, mainly to provide tools for the early diagnosis of neurodegenerative diseases (see Carnero-Rosell et al.^43^). The cosmic density field represents a multivariate statistical problem, where each voxel in the three-dimensional grid provides statistical information about the cosmic volume^44^. Similarly, in the context of brain MRI, each voxel represents a location within the brain’s anatomy, allowing us to capture intricate spatial distributions. By applying two- and three-point statistics to brain MRI data in Fourier space, we transform this complex three-dimensional information into a more interpretable format, such as the clustering amplitude of a triangle configuration given two sides as a function of the subtended angle. This kind of data can be trivially converted into time-series data for sonification. The combination of two- and three-point statistical analysis allows for a robust framework that captures the essential structural characteristics of the data, opening up new avenues for scientific inquiry and improving data accessibility. Details on the sonification method can be found in the methods section (see Figure 1).

## Sonification of bispectra from magnetic resonance imaging data

Based on the OASIS-3 database (see methods section), we examine the bispectrum configuration of the MRI data from healthy subjects in different age ranges, which exhibits complex behavior by integrating information from both the two- and three-point statistics, commonly referred to as the reduced bispectrum. For a comprehensive introduction to higher-order statistics and reduced bispectra in particular, we refer to Carnero-Rosell et al.^43^ and references therein. Specifically, we focus on the reduced bispectrum configuration Q019036 with wave numbers $k_{1}=0.19$ and $k_{2}=0.36{\;\text{mm}}^{-1}$ (see figure 3). Using this particular configuration as a biomarker achieves an averaged mean absolute error (MAE) of approximately 4.7 years for predicting age between ~40 and 100 years. We employed a Random Forest classifier^45^ using a leave-one-out cross-validation (LOO-CV) technique^46^, ensuring that each subject’s estimate was independent of the training sample. Age-regression with neural networks can improve the MAE to 4.2 years with the same bispectrum configuration^43^. Importantly, exploring additional bispectrum configurations is beneficial, as it allows capturing information across multiple spatial scales (both large and small), thus enabling a more comprehensive characterization of brain aging. We selected the configuration above as a challenging example since it spans a wide range of values yet demonstrates subtle internal variations, which are particularly difficult to represent effectively through sonification.

**Figure 2.**
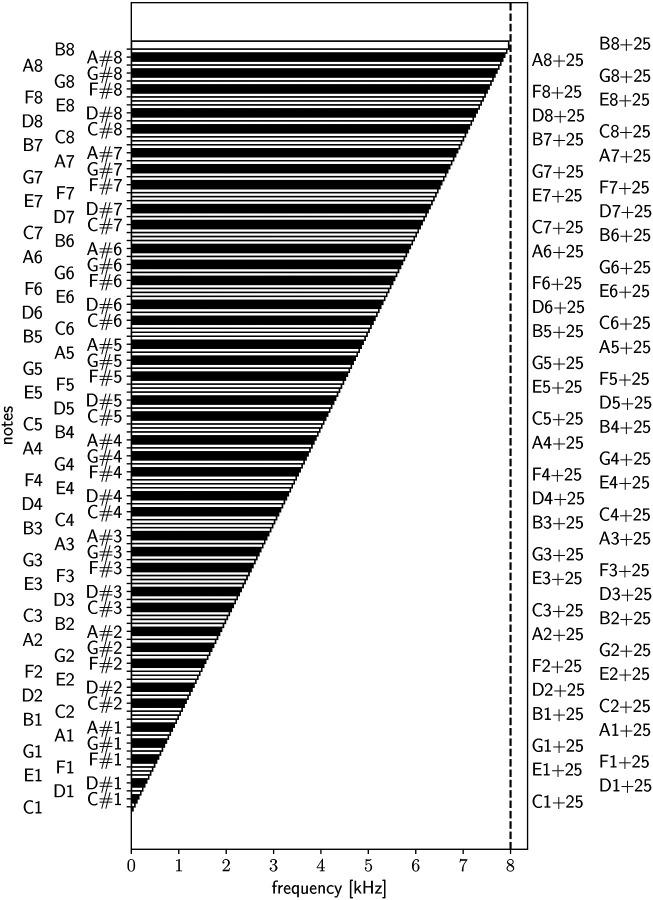
Sonification piano: notes used in this study covering the frequency range within the sensitivity of adult humans (20–30 yrs: ~16, 40 yrs: ~14, 50 yrs: ~12, 60 yrs: ~10, 70 yrs: ~8 kHz). Regular notes (${\text{C}}_{i},{\text{D}}_{i},{\text{E}}_{i},{\text{F}}_{i},{\text{G}}_{i},{\text{A}}_{i},{\text{B}}_{i}$ for different octaves i ranging from 1 to 8) have been split into two columns (first two on the left) for visualisation purposes. The same has been done with quarter tones (indicated by +25) on the right. Semitones are indicated in the third column on the left. The piano convention of black keys for semitones is adopted. We consider sonification cases with and without quarter tones depending on the bispectrum configuration and the desired accuracy.

**Figure 3.**
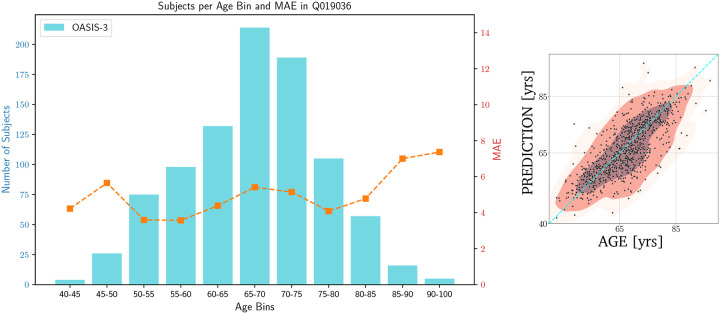
On the left: age distribution for the OASIS-3 864 MRI sessions dataset reduced by Carnero-Rosell et al.^43^ with the corresponding MAE at different AGE bins according to Random Forests classification based solely on the Q019036 bispectrum configuration. On the right: corresponding age regression.

**Figure 4.**
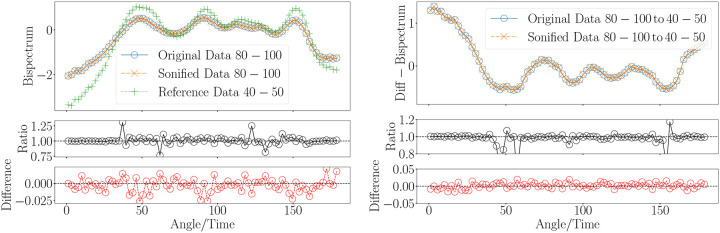
Sonification of bispectra from human MRI, using an age group of 80–100 years as an example. The original data and the corresponding inverse mapping after sonification are displayed, with the bispectrum for the 40–50 age group included as a reference. The ratios and differences between the original and the inverse-mapped sonified signals are also presented.

Our goal is to assign distinct musical notes and envelope to the significant variations in the bispectrum signal, allowing for a smooth auditory representation of its transitions, while ensuring the signal remains within the audible frequency range for adult human listeners. This task becomes challenging, when realising the large bispectrum range differences between the younger and older groups. To achieve this, we have selected a range of notes spanning eight octaves, including semitones and, when necessary, quarter tones, covering frequencies from a few tens of Hertz up to approximately 8 kHz. While further tone subdivisions are possible, they would likely compromise auditory distinguishability. The frequency range was chosen to encompass both the subtle and significant variations in the bispectrum signal, ensuring that the transitions are captured smoothly and remain within the audible range for adult humans. For more details, see Figure 2 and methods section, where the specific frequency mapping and note selection are discussed in depth. This setup has allowed us to accurately sonify the bispectra across different age groups while maintaining a consistent amplitude range. Technical details of the sonification procedure can be found in the methods section. We based the amplitude range on the group exhibiting the largest variations, specifically the youngest cohort in our sample: 40–50 years (see left panel in Figure 4). By standardizing the amplitude in this way, we ensure that the sonification of bispectrum variations remains comparable across age groups, providing a clear auditory representation of the differences in the bispectrum signal across the age spectrum.

To assess the accuracy of the sonification procedure, we adopt an information-theoretic approach to evaluate how well the original signal can be recovered after undergoing sonification (see methods section). This process involves discretizing the continuous bispectrum signal into integer values, and the challenge lies in determining how much information is lost in this transformation. By analyzing the fidelity of the recovered signal compared to the original, we can quantify the effectiveness of the sonification process and ensure that the discretization does not significantly degrade the essential features of the bispectrum signal. We treat the originally measured bispectrum from MRI data and its sonified version in two ways: first, as a “ground truth” function Y and a “modeled” function $\overset{ˆ}{Y}$, and second, as their corresponding probability distribution functions P and Q, respectively. The accuracy of the sonification method is then evaluated through metrics defined between Y and $\overset{ˆ}{Y}$, or between P and Q, allowing us to measure the fidelity of the sonified signal compared to the original.

During the reconstruction study, we found that the discretization step can introduce systematic biases, which could affect the accuracy of the sonified signal. However, applying an appropriate rounding procedure, can effectively mitigate these biases. While the differences between distant age groups are perceptible in the sonifications, distinguishing between closer age groups becomes more challenging. For instance, it is difficult to differentiate between the 40–50 and 50–60 year age groups. This is due to the more subtle variations in the bispectrum signal for these adjacent age ranges, which are less pronounced in the auditory representation than the differences observed between more distant groups.

To address this challenge, we propose sonifying the differences between different age groups’ bispectrum signals, rather than the signals themselves (see right panel in Figure 4). This approach emphasizes the subtle variations that might otherwise be hard to distinguish. Additionally, to further enhance the sonified output, we introduce a normalization factor (less than 1) that amplifies these differences, making them more perceptible. This normalization highlights the variations and ensures that the signal remains within a consistent and manageable auditory range. Another way to enhance the sonified representation of the bispectrum signal involves applying a nonlinear transformation that stretches or compresses the dynamic range of the data. This technique improves the dynamic representation of the sonified signal by amplifying or reducing the variations in amplitude, making subtle differences more perceptible, without altering the overall structure or rank order of the data. Applying this technique we have found ways to reduce the ratio deviations (see Figure 10).

By applying this approach to the differences between the age groups 40–50 and 50–60, 40–50 and 70–80, as well as 40–50 and 80–100, we observe surprisingly similar signal shapes across these comparisons, although the amplitudes differ, indicating a potential homologous brain aging. However, the amplitude variation can be effectively managed with adaptive normalization, ensuring that the differences remain perceptible while maintaining a consistent volume range for the sonified signals.

**Figure 5.**
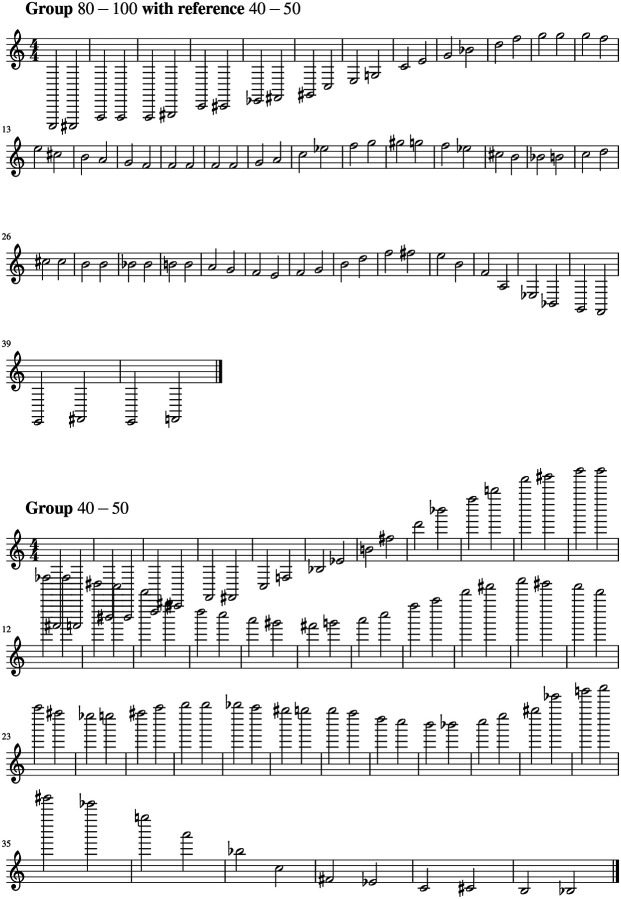
Scores corresponding to (top:) the age group of 80 to 100 using as the reference the group of 40 to 50 to set the range; and (bottom:) the age group of 40 to 50.

**Figure 6.**
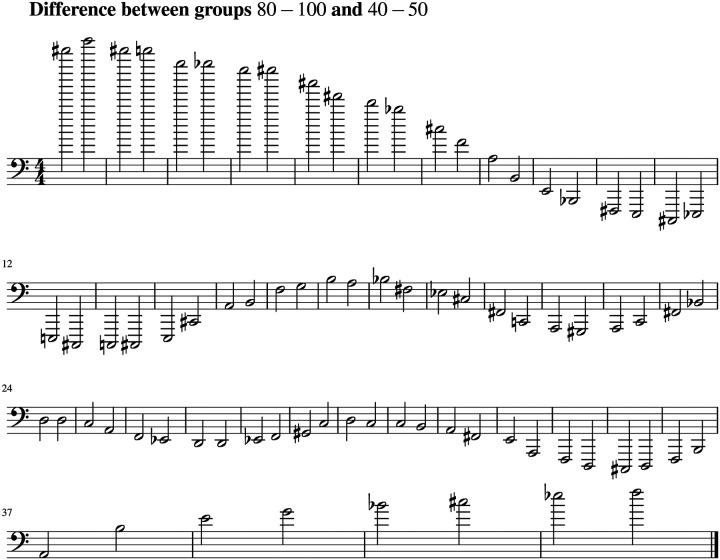
Scores corresponding to the difference between the age group of 80 to 100 with the group of 40 to 50.

Based on the results shown in Figure 4 we write the corresponding scores with tones and semitones. The scores corresponding to the sonified bispectrum signals for the 80–100-year and 40–50-year age groups are shown in Figure 5. The score in Figure 6 highlights the structured patterns in the differences between these age groups, emphasizing the informational content underlying these variations.

## Discussion

In this work, we investigated the use of sonification of higher-order summary statistics as a method for characterizing complex multidimensional patterns within one-dimensional time series.

In particular, we explored sonification of bispectrum signals derived from MRI data, with the goal to provide an intuitive and effective auditory representation of complex higher-order statistics, such as the reduced bispectrum. This method holds promise for diagnostic applications, particularly in detecting early signs of dementia and monitoring deviations from expected bispectrum patterns for specific age groups.

We have introduced a large notes array, offering a densely distributed set of notes to capture and sonify subtle differences in the bispectrum signal. This array spans a wide frequency range, allowing for the representation of both fine details and broader variations in the data. By ensuring a sufficient number of notes across the audible spectrum, we can maintain precision in the sonification process, making even minor deviations perceptible while preserving the overall integrity of the signal. This approach ensures that the full range of bispectrum variations is accurately conveyed through sound.

Additionally, we have demonstrated how to enhance the bispectrum signal for sonification through appropriate normalization, ensuring that amplitude variations remain within a manageable and perceptible range. Furthermore, we have introduced nonlinear rank-ordered transformations to reduce deviations, allowing for dynamic adjustments that stretch or compress the signal’s range while preserving the relative structure and order of the data. These techniques collectively improve the accuracy and clarity of the sonified signal, making subtle differences more audible and enhancing the overall effectiveness of the sonification process.

The examples shown in this study also demonstrate the potential of sonifying bispectrum difference signals for acoustic diagnostics, particularly identifying deviations from a reference bispectrum signal for a specific age group. By focusing on the differences between bispectrum signals, subtle variations that might indicate cognitive changes can be identified, providing a novel and potentially effective method for early detection through auditory analysis. The extension of this study to the sonification of bispectrum signal configurations across multiple scales can significantly enhance and deepen diagnostic analysis. While this approach shows promise, a thorough study investigating its full potential for medical diagnostics, particularly in neurodegenerative diseases, is left for future work.

One may investigate the potential relationship between the sonification of brain MRI and the process of speech production, where the brain plausibly transforms complex, multidimensional signals (i.e., thoughts) into a linear sequence of speech sounds.

The sonification technique presented here can generally be used to analyse any multi-dimensional data from any field of research, such as, in the field of neuroscience: functional MRI, positron emission tomography, or even functional connectivity maps derived from EEG and MEG, which makes it a very promising tool in the field.

## Methods

### Input data

The data considered in this study was prepared by Carnero-Rosell et al.^43^ within the Cosmic Brain project based on the OASIS-3 database^47^.

All MRI data was collected through the Knight Alzheimer Research Imaging Program at Washington University in St. Louis, MO, USA. Some of the MRI data was collected on a Siemens Vision 1.5T, while the majority of the scans came from two different versions of a Siemens TIM Trio 3T (Siemens Medical Solutions USA, Inc). Participants were lying in the scanner in a supine position, head motion was minimized by inserting foam pads between the participant’s head and antenna coil, and for some participants a vitamin E capsule was placed over the left temple to mark lateralization. A 16 channel head coil was used in all scans. Although a variety of different structural and functional imaging protocols are included in the OASIS dataset such as FLAIR, DTI and ASL, here we focused on the T1w scans. The T1w images were acquired using a 3DMPRAGE protocol TI/TR/TE: 1000/2400/3.08 ms, flip angle = 8°, resulting in 1 mm isotropic voxels.

Data were provided by OASIS-3: Longitudinal Multimodal Neuroimaging: Principal Investigators: T. Benzinger, D. Marcus, J. Morris; NIH P30 AG066444, P50 AG00561, P30 NS09857781, P01 AG026276, P01 AG003991, R01 AG043434, UL1 TR000448, R01 EB009352. AV-45 doses were provided by Avid Radiopharmaceuticals, a wholly owned subsidiary of Eli Lilly.

### Sonification frequency range

The first note C_1_ has a frequency of 32.702 Hz, while the last regular note B_8_ has a frequency of 7902.13 Hz.

In case we include quarter tones, we need to make additional calculations. The 12-tone equal temperament system divides an octave into 12 equal parts, where each part corresponds to a semitone. In this system, the frequency of a note increases by a factor of 2 when going up by an octave. Moving up by one semitone corresponds to multiplying the frequency by 2^1/12^, since there are 12 semitones in an octave. The cent is a unit used to measure musical intervals, specifically fractions of semitones. There are 100 cents in a semitone.

To calculate the frequency change for a given number of cents, we use the formula $2^{n/1200}$, where n is the number of cents. For a quarter tone, which is 25 cents (half of a semitone), we substitute n=25 into the formula: $f_{\text{factor}}=2^{25/1200}$. To calculate the frequency of B8 + 25 (25 cents above B8), we have to multiply the original frequency by the quarter tone factor: $f_{\text{B}8+25}=7902.13\times 2^{25/1200}=7961.09\;\text{Hz}$.

Hence, the total frequency range we are considering when including quarter tones spans from 32.702 to 7961.09 Hz, just below the typical upper sensitivity limit of 8 kHz for humans of about 70 yrs. This should be considered when targeting older age groups.

### Methodology Overview

**Figure 7.**
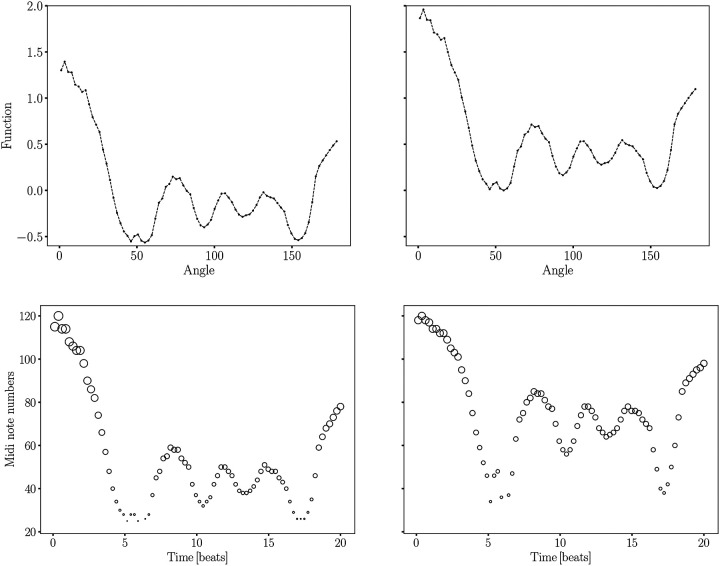
Sonification procedure across four panels: The upper-left panel shows the original signal with each data point represented by a small circle. In this example, the difference of the reduced bispectra (configuration $k_{1}=0.19$ and $k_{2}=0.36$) for age groups: 80–100 and 40–50 is shown. In the upper-right panel, the minimum value is subtracted from the signal. The lower-left panel illustrates the signal discretization process, additionally applying a linear mapping to convert the signal into the range of MIDI values that are perceptible to adult human listeners. Finally, the lower-right panel demonstrates the effect of incorporating a nonlinear rank-ordered transformation with a power-law of exponent $f_{\text{exp}}=0.45$. The velocity (volume) of the sonified signal is represented through the size of the circles. The normalization is one.

**Figure 8.**
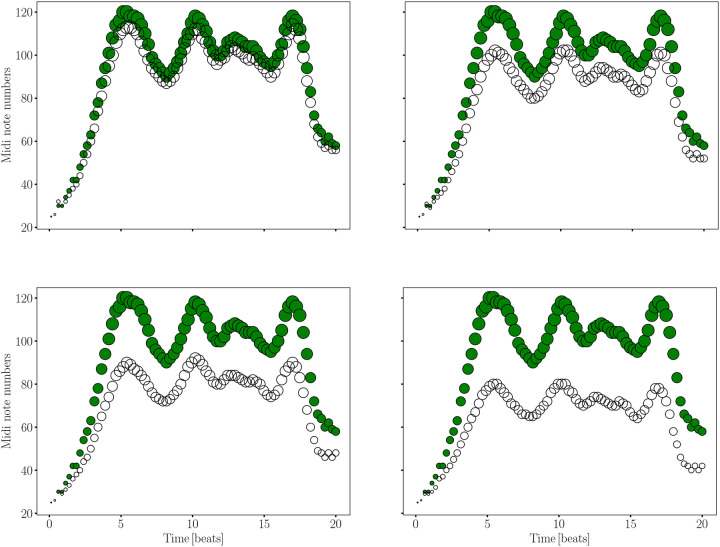
Similar sonification procedure to Figure 7, but showing relative reduced bispectra relations (configuration $k_{1}=0.19$ and $k_{2}=0.36$). The different panels show the discretized signal for the 50–60 (upper-left), 60–70 (upper-right), 70–80 (lower-left), 80–100 (lower-right) age group within the range of the reference 40–50 years group mapped to the corresponding MIDI values. The filled circles stand for the reference bispectrum.

**Figure 9.**
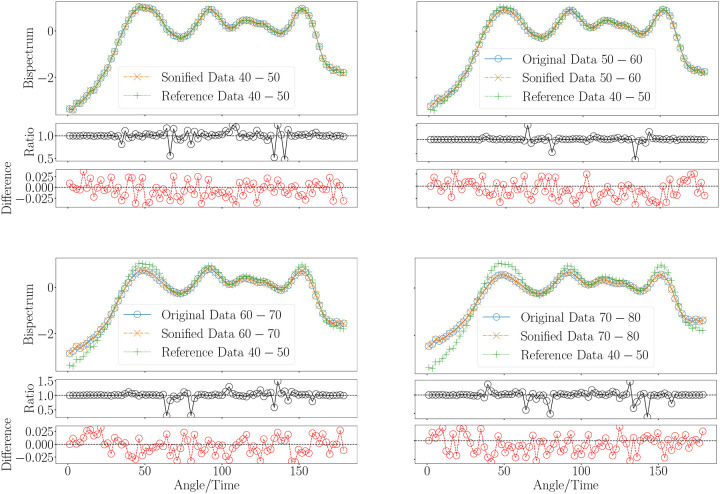
Sonification of reduced bispectra (configuration $k_{1}=0.19$ and $k_{2}=0.36$) from human brain magnetic resonance imaging for different ages. The original data and the corresponding inverse mapping after sonification are indicated for each age range.

**Figure 10.**
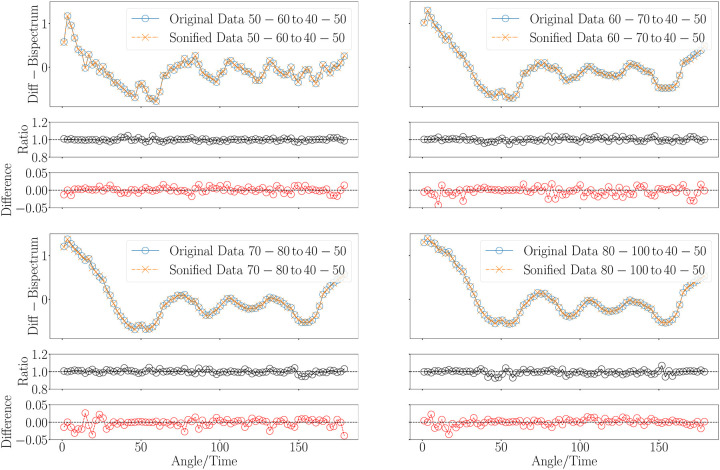
Sonification of difference between reduced bispectra (configuration $k_{1}=0.19$ and $k_{2}=0.36$) from human brain magnetic resonance imaging for different ages groups with respect to the youngest age group in our sample (40–50 yrs). Upper-left panel: $c_{\text{norm}}=0.25,f_{\text{exp}}=0.7$; upper-right panel: $c_{\text{norm}}=0.5,f_{\text{exp}}=0.35$; lower-left panel: $c_{\text{norm}}=0.75,f_{\text{exp}}=0.35$; lower-right panel: $c_{\text{norm}}=1,f_{\text{exp}}=0.45$.

This methodology section describes the steps involved in converting bispectrum MRI data into sonifiable MIDI sequences. The sonification steps can be seen in Figure 7. Figure 8 shows the sonification procedure to present relative signals with respect to a reference one which determines the range. We can see from the upper-left panel of Figure 8 (corresponding to the bispectra shown in Figure 9), that the differences between the 40–50 and 50–60 years group are very tiny. In such cases it seems more adequate to focus on the sonification of the differences. We demonstrate in Figure 10 how those differences can be enhanced applying the method explained below.

#### 1. MRI data preparation:

The first step involves obtaining the MRI brain intensity map on a mesh. This map is represented in three dimensions, with each voxel containing an intensity value corresponding to a specific brain location. These values provide the base data for subsequent analysis.

#### 2. Statistical analysis:

To capture the structural complexity of the brain, we compute two-point and three-point correlation functions in Fourier space.

Details on these computations can be found in Carnero-Rosell et al.^43^ and references therein.

#### 3. Bispectrum normalization:

The data is normalized to ensure consistency across datasets: $y_{i}^{\prime }=\frac{y_{i}}{c_{\text{norm}}}$, where $c_{\text{norm}}$ is a predefined normalization constant. This step ensures that intensity values are comparable and prevents skewing due to extreme values.

#### 4. Bispectrum linear mapping to a positive definite range:

We define the linear mapping function “map_value”:

$${\tilde{y}}_{i}=\text{map}\_\text{value}\left(y_{i}^{\prime },y_{\text{min}}^{\prime },y_{\text{max}}^{\prime },y_{\text{min}},y_{\text{max}}\right)\;=y_{\text{min}}+\frac{\left(y_{i}^{\prime }-y_{\text{min}}^{\prime }\right)}{\left(y_{\text{max}}^{\prime }-y_{\text{min}}^{\prime }\right)}\cdot \left(y_{\text{max}}-y_{\text{min}}\right)$$

The normalized data is linearly mapped to a positive finite range $\left[y_{\text{min}},y_{\text{max}}\right]$ to facilitate MIDI conversion:

$${\tilde{y}}_{i}=\text{map}\_\text{value}\left(y_{i},y_{\text{min}},y_{\text{max}},y_{\text{min}},y_{\text{max}}\right)$$

#### 5. Time linear mapping to a positive definite range:

The subtended angles from triangle configurations are converted to a time line:

$$t_{i}=\text{map}\_\text{value}\left(\theta_{i},\theta_{\text{min}},\theta_{\text{max}},0,\text{duration\_beats}\right)$$

We are choosing, per default, a duration of 2 seconds, a duration_beats of 20, and a beats per minute (bpm) of 60.

#### 6. Nonlinear rank-ordered transformation:

To enhance perceptibility, the data is then exponentiated by a factor $f_{\text{exp}}:y_{\text{map}}={\tilde{y}}^{f_{\text{exp}}}$.

This process stretches $\left(f_{\text{exp}}>1\right)$ or compresses $\left(f_{\text{exp}}<1\right)$ the dynamic range of the data, improving the sonification’s dynamic representation, while preserving the structure and rank order. For $f_{\text{exp}}=1$ we have $y_{\text{map}}=\tilde{y}$.

#### 7. Sonification: mapping to MIDI notes:

The transformed intensity values are mapped to MIDI^1^ notes using the function:

$$\text{MIDI}\;\text{value}=69+12{\;\text{log}}_{2}\left(\frac{f}{440}\right)$$

where f is the frequency corresponding to the intensity value.

The MIDI note number 69 corresponds to the musical note A4, which is standardized at a frequency of 440 Hz (often referred to as “concert pitch”). Quarter tones and octave shifts are adjusted accordingly. The MIDI note pitch is derived by discretizing the intensity range into predefined musical notes (see Figure 2):

$${\text{index}}_{\text{note}}=\text{round}\left(\text{map\_value}\left(y_{\text{map}},y_{\text{min}}^{\prime },y_{\text{max}}^{\prime },0,n_{\text{notes}}-1\right)\right),$$

where the range is adjusted, in the relative representation case (see Figure 8), to a reference signal covering the largest range:

$$y_{\text{min}}^{\prime }=\text{min}\left(y_{\text{map}}\right)\times \text{scale\_factor}\;y_{\text{max}}^{\prime }=\text{max}\left(y_{\text{map}}\right)\times \text{scale\_factor},$$

and

$$\text{scale\_factor}=\frac{\text{max}\left(y_{\text{ref}}\right)-\text{min}\left(y_{\text{ref}}\right)}{\text{max}\left(y_{\text{map}}\right)-\text{min}\left(y_{\text{map}}\right)}.$$

The scale factor is set to one when computing differences. With the index we obtain the MIDI notes: ${\text{MIDI}}_{\text{value}}[{\text{index}}_{\text{note}}]$. The volume (or velocity) of each note is assigned on a suitable range:

$$\text{vel}=\text{round}\left(\text{map}\_\text{value}\left(y_{\text{map}},\text{min}\left(y_{\text{map}}\right),\text{max}\left(y_{\text{map}}\right),{\text{vel}}_{\text{min}},{\text{vel}}_{\text{max}}\right)\right)$$

where the velocity lies within the range 65 ≤ vel ≤ 110 to ensure perceptibility. The term “velocity” in MIDI was chosen to represent the intensity of a note, but it doesn’t refer to the literal speed of a note’s sound. Instead, it refers to how quickly the key is pressed (from a physical perspective) and how that is mapped to the loudness or dynamic quality of the note.

The reduced bispectra sonification can be seen in Figures 9 and 10. The latter shows the application of the normalization and the nonlinear transformation. The nonlinear transformation enhances the representation of features that exhibit minimal variations within a signal that spans a wide range of values. We can see in the lower-left panel of Figure 7, how some bispectrum bins acquire the same value in the sonfication process. This problem is not present in the lower-right panel where $f_{\text{exp}}=0.45$ was applied. As a consequence of this transformation the ratio plots in the top and bottom panels of Figure 10 display a flatter behaviour than in Figure 4.

### Information loss

To assess the accuracy of the sonification, the information loss is calculated by transforming the MIDI values back into the original bispectrum space: ${\tilde{y}}_{\text{inverse}}=\text{map}\_\text{value}\left({\text{MIDI}}_{\text{value}},{\text{MIDI}}_{\text{min}},{\text{MIDI}}_{\text{max}},y_{\text{min}},y_{\text{max}}\right)$.

This includes reversing the exponentiation (for $f_{\text{exp}}\neq 1$): $y_{\text{inverse}}={\tilde{y}}_{\text{inverse}}^{1/f_{\text{exp}}}$.

Information loss is measured using, e.g., the normalised mean squared error (NMSE):

$$\text{NMSE}=\frac{\frac{1}{n}\sum_{i=1}^{n}{\left(y_{i}-y_{\text{inverse,}i}\right)}^{2}}{\frac{1}{n-1}\sum_{i=1}^{n}{\left(y_{i}-\overset{‾}{y}\right)}^{2}}.$$

For a more complete metric analysis see Table 1 applied to the reduced bispectrum for both absolute and differences sonification. These metrics provide insights into the accuracy of the sonification and guide any necessary adjustments.

Depending on the metric, we will treat the data as a function (Y) or as a PDF (P):

Given $\overset{ˆ}{Y}=\text{ground}\;\text{truth}\;\text{data}\;\text{set}$, Y=modeldataset,

- Make the data positive definite:
$$\text{if}\;\text{min}(Y)<0,\;\text{then}\;\text{set}\;Y=Y-\text{min}(Y),\;\text{if}\;\text{min}(\overset{ˆ}{Y})<0,\;\text{then}\;\text{set}\;\overset{ˆ}{Y}=\overset{ˆ}{Y}-\text{min}(\overset{ˆ}{Y}),$$
- Construct probability distributions:
$$P=\frac{Y}{\sum Y},\;Q=\frac{\overset{ˆ}{Y}}{\sum \overset{ˆ}{Y}}.$$

The metrics in Table 1 were obtained with the inclusion of quarter tones. Results without quarter tones were worse by about an order of magnitude in the NMSE.

## Figures and Tables

**Figure 1.**
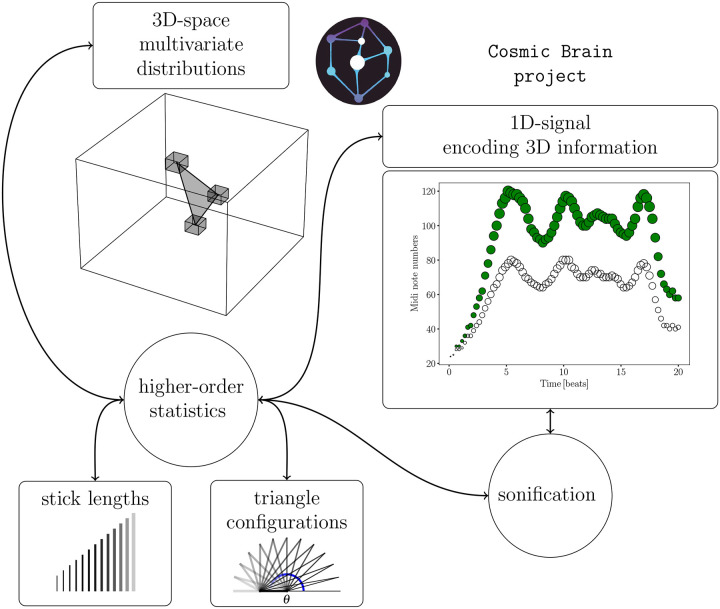
This diagram illustrates the sonification method for three-dimensional data, utilizing higher-order statistical analysis in Fourier space. The two-point statistics are represented by sticks of varying lengths, which connect pairs of voxels (in the configuration space analog), indicating their respective intensities from the MRI scan. The three-point statistics are depicted through different triangle configurations, where two fixed side lengths are considered with varying subtended angles to connect three voxels. A one-dimensional function is derived by combining these statistical measures, which can then be directly sonified.

**Table 1.** Metrics and their mathematical formulae computed for different age groups corresponding to the reduced bispectra (configuration $k_{1}=0.19$ and $k_{2}=0.36$). The ideal case is indicated within brackets, e.g., [→ 0] or [→ 1]. “ABS” stands for sonification of absolute bispectra, and “DIFF” stands for difference between bispectra of a group and a reference one of 40–50.

Statistical Measure Name (SMN)	Mathematical Formula	Age Group								
Mean	$\overset{‾}{y}=\frac{1}{n}\sum_{i=1}^{n}y_{i}$	40–50	50–60		60–70		70–80		80–100	
Variance	$Var(Y)=\frac{1}{n-1}\sum_{i=1}^{n}{\left(y_{i}-\overset{‾}{y}\right)}^{2}$	ABS	ABS	DIFF	ABS	DIFF	ABS	DIFF	ABS	DIFF
Mean Absolute Error [→0]	$\text{MAE}(Y,\overset{ˆ}{Y})=\frac{1}{n}\sum_{i=1}^{n}\left|y_{i}-{\overset{ˆ}{y}}_{i}\right|$	0.0026	0.0162	0.0015	0.0003	0.0046	0.0104	0.0064	0.0078	0.0064
Mean Squared Error [→0]	$\text{MSE}(Y,\overset{ˆ}{Y})=\frac{1}{n}\sum_{i=1}^{n}{\left(y_{i}-{\overset{ˆ}{y}}_{i}\right)}^{2}$	0.0004	0.0004	0.0000	0.0169	0.0000	0.0001	0.0001	0.0001	0.0001
Root Mean Squared Error [→0]	$\text{RMSE}(Y,\overset{ˆ}{Y})=\sqrt{\text{MSE}}$	0.0194	0.0192	0.0020	0.0169	0.0062	0.0127	0.0089	0.0102	0.0088
Normalized MSE [→0]	$\text{NMSE}(Y,\overset{ˆ}{Y})=\frac{\text{MSE}}{\text{Var}(Y)}\times 100[\%]$	0.0255	0.0274	0.0571	0.0275	0.0832	0.0215	0.0587	0.0199	0.0292
Relative MSE [→0]	$\text{ReMSE}(Y,\overset{ˆ}{Y})=\frac{\text{MSE}}{{\bar{y}}^{2}}\times 100[\%]$	0.4138	0.3510	0.0132	0.2763	0.0351	0.1734	0.0303	0.1422	0.0217
Signal-to-Noise Ratio [→≫1]	$\text{SNR}(Y,\overset{ˆ}{Y})=\frac{\text{Var}(Y)}{\text{MSE}}$	3924.6	3648.1	1750.3	3642.7	1202.4	4649.4	1704.2	5017.8	3420.7
R-squared [→1]	$R^{2}(Y,\overset{ˆ}{Y})=1-\frac{\sum_{i=1}^{n}{\left(y_{i}-{\overset{ˆ}{y}}_{i}\right)}^{2}}{\sum_{i=1}^{n}{\left(y_{i}-\overset{‾}{y}\right)}^{2}}$	0.9997	0.9997	0.9994	0.9997	0.9993	0.9998	0.9994	0.9998	0.9997
Explained Variance [→1]	$\text{EVAR}(Y,\overset{ˆ}{Y})=1-\frac{\text{Var}(Y-\overset{ˆ}{Y})}{\text{Var}(Y)}$	0.9998	0.9998	0.9994	0.9997	0.9992	0.9998	0.9992	0.9998	0.9997
Cosine Similarity [→1]	$\text{CS}(P,Q)=\frac{\sum_{i=1}^{n}p_{i}\cdot q_{i}}{\sqrt{\sum_{i=1}^{n}p_{i}^{2}}\cdot \sqrt{\sum_{i=1}^{n}q_{i}^{2}}}$	1.0000	1.0000	0.9999	1.0000	0.9999	1.0000	0.9999	1.0000	0.9999
Kullback-Leibler Divergence [→0]	$D_{\text{KL}}(P,Q)=\sum_{i=1}^{n}p_{i}\;\text{log}\;\frac{p_{i}}{q_{i}}$	0.0000	0.0000	0.0001	0.0000	0.0001	0.0000	0.0001	0.0000	0.0001
Cross-Entropy [→0]	$H(P,Q)=-\sum_{i=1}^{n}p_{i}\;\text{log}\;q_{i}$	0.0535	0.0534	0.0533	0.0534	0.0521	0.0534	0.0516	0.0534	0.0504
Total Variation Distance [→0]	$\text{TVD}(P,Q)=\frac{1}{2}\sum_{i=1}^{n}\left|p_{i}-q_{i}\right|$	0.0024	0.0024	0.0043	0.0026	0.0066	0.0022	0.0062	0.0021	0.0053
Hellinger Distance [→0]	$D_{\text{H}}(P,Q)=\sqrt{\frac{\sum_{i=1}^{n}{\left(\sqrt{p_{i}}-\sqrt{q_{i}}\right)}^{2}}{2}}$	0.0027	0.0026	0.0042	0.0029	0.0060	0.0025	0.0055	0.0022	0.0052

## Footnotes

1 MIDI (Musical Instrument Digital Interface) is a technical standard that describes a communication protocol, digital interface, and electrical connectors that connect a wide variety of electronic musical instruments, computers, and related audio devices for playing, editing, and recording music^48,49^.
